# Medicine as a Profession

**Published:** 1908-09-05

**Authors:** 


### The Hospital

A Journal of

The Medical Sciences and Hospital Administration.

New Series. No. 78, Vol. III. [No. 1150, Vol. XLIV.] SATURDAY, SEPTEMBER 5, 1908.

### EDUCATIONAL NUMBER.

### MEDICINE AS A PROFESSION.

"Let parents choose betimes the vocations and courses they mean their children should take; for then they are most flexible; and let them not too much apply themselves to the dispositions of their children as thinking they will take best to that which they have most mind to. It is true that if the affection or aptness of the children be extraordinary it is good not to cross it; but generally the precept is good, *optimum elige, suave et facile illud faciet consuetudo.*"

So says Bacon, and it is without doubt wise advice; for with the generality of boys and girls the attractions and repulsions of various professions are not fixed and enduring, but transitory and dictated by momentary currents of feeling. During the period of adolescence, at which the choice of life's work has commonly to be made, the human economy (and particularly its mental and moral components) is labile and unstable, apt to receive passing impressions and as apt to lose them. Reasoned judgment is not to be looked for, while the response to emotional dictates is active and often violent, yet not abiding. It is clear, therefore, that in the average case the parent or guardian must supply the balanced judgment which can survey with detachment the pros and cons of the alternatives which offer. There are of course exceptions, as Bacon says: children who, from an early age, evince a bent so pronounced and continued that opposition to it involves a considerable risk of disaster. This bent may be positive or negative towards any given avocation, and in either case demands an equal regard, provided it be sufficiently pronounced and permanent. It may show itself positively in the class of hobbies chosen for the occupation of leisure, or negatively in a patent aversion to all employments or studies of a particular order.

Indications of this kind are of such palpable moment in the choice of a career that it is not necessary to labour the need for attending to them; but in a large majority of cases they do not exist to a degree sufficiently pronounced to entitle them to such paramount respect. It remains to be said, however, that neither the absence of a repulsion nor a positive attraction will justify the choice of medicine as a profession for children whose antecedents are badly tainted by psychoses or grave neuroses, particularly when they themselves show signs of nervous instability. For the profession of medicine involves much acquaintance with grief and urgent responsibilities to be undertaken at a moment's notice, and these are things to which persons of highly nervous and emotional temperament cannot be submitted with impunity. For them a life of even tenor is far more likely to be a life of happiness.

So much for the exceptional cases. They are rare, of course, but the misunderstanding of them leads from time to time to failure or even tragedy. For the common run of healthy boys most professions may be relied upon to produce interest and happiness in proportion to the success achieved, custom, as says Bacon's adage, making the employment pleasant and easy even in the absence of any instinctive attraction. The rewards of every profession come under two heads, firstly those which depend upon the intrinsic pleasure of the occupation, and secondly those associated with financial success and material dignity. These considerations will of course appeal with varying force to varying types of men; but it may be said without cant that no one can afford to neglect either of them. Let us then consider briefly how the practice of medicine stands in relation to them both.

As regards the interest of the work we may assert without fear of contradiction that the life of a doctor need yield to none in the matter of sustained and varied interest. His daily business is the study of human nature: and this not the human nature which is commonly presented to the world decorously attired in forms and conventions, but human nature unadorned—full of puzzling contradictions, elusive and of infinite variety. From the very commencement of his studies the student deals with subjects capable of being illuminated by ocular experiment, subjects demanding mechanical manipulations entertaining in themselves and affording a far more pleasant path to the acquisition of knowledge than that which, as is the case with other learned professions, lies almost solely in the pages of heavy treatises. Throughout his career, whether it be conducted in part at a university or entirely at one of the great medical schools, the student enjoys a position not unlike that of an undergraduate at one of the universities. He is a member of a body corporate, with ample scope for the formation of lasting friendships and abundant recreations both for body and mind, and it is common to find that the five years of apprenticeship form an epoch which remains an abiding memory of pleasure in the minds of medical men. At its conclusion in a majority of cases a man has formed his determination concerning the branch of practice to which he is most attracted, and can for the most part, though with certain qualifications, follow it. There is no paucity of alternatives. This is an age of specialisations, for the advances of science have extended the field of medicine almost beyond belief, and it is impossible for any man to keep a place in the front rank all along the line. He may choose then from the following considerable list. If his aptness lies rather with the scientific than the practical side of medicine there are open to him academic positions in the various schools of medicine, lectureships and, later, professorships of subjects such as anatomy and physiology, bacteriology or pathology. If he incline rather to the practical side his opportunities are yet more abundant: general practice at home or abroad; the medical services of the Army, the Navy, the Colonial department, and the Indian Government; work in asylums and fever hospitals and sanatoria for consumption; the municipal public health appointments, and finally all the special branches of practice, medicine, surgery, gynæcology, otology, ophthalmic surgery, laryngology, dermatology, electro-therapeutics, and not a few besides.

It will go hard then with a student of medicine if, when he has qualified himself to practise, he cannot find a suitable outlet for his professional energies, as far at least as the interest of it is concerned. It must be confessed that the material side of the business is not quite so attractive, though we fancy a good deal of unnecessary pessimism has been lavished upon it. Let us first of all observe—and this must be kept constantly in view in the contemplation of this aspect of the matter before us—that a qualification to practise medicine is in a sense so much perpetual capital to the man who possesses it. It is true that its possession does not in itself imply interest upon it, but interest can always be obtained by labour, and the capital cannot be dissipated except by ill-health or evil living. In this respect medicine has a distinct advantage over the bar for example: for the briefless barrister may well starve, but the qualified practitioner of medicine need never want for a livelihood so long as he preserves his health and his good name. The livelihood it is true may be exiguous, but it is always at command, and this cannot be said with equal justice of any other learned profession. As regards the amassing of money, medicine stands indubitably low, and the man whose ambitions are bounded by nothing but money-bags had best avoid it. There is no lack of reasons for this state of affairs, but the prime one is that medicine and charity are in the nature of things allied, and a doctor has to do a good deal of work for nothing or next to nothing, while the style of living demanded of him by convention makes large drafts upon his earnings. None the less, the man who commands intelligence and application in average quantities will find in the practice of medicine an occupation which in general terms compares favourably with other learned professions as regards monetary return, while in interest, as we have said, it cannot be surpassed.

We have spoken above of certain qualifications which limit the freedom with which a man can choose the branch of medicine he desires to pursue. Thus for the man with no capital at all the public medical services offer the attraction of an immediate living wage, a regular salary, if not a large one, a pleasant life, and the prospect of a pension. To the man with some capital there is a wider choice; for if he can afford to face lean years at the commencement, the practice of the various specialities is open to him, while if he prefers to invest his money at once he may do so by purchasing a share in a private practice of general medicine. The successful pursuit of specialities can hardly be achieved except by the aid of appointments to hospitals, particularly those to which medical schools are attached. In consequence the competition for these posts is extremely keen and the periods of waiting for vacancies often protracted. During these years of waiting the aspirant must busy himself with the accumulation of knowledge in his speciality by the tenure of unpaid appointments at minor hospitals, and of minor teaching appointments in the school of the hospital to which he aspires. It is in these years that the possession of some capital does so much to smooth the way and relieve the anxieties otherwise arising from lack of pence. Of the specialities themselves there are some which begin to bring in money at an earlier date than others. It would appear that the practice of consulting medicine holds the unenviable position of being that branch whose rewards are most postponed; for this is a class of practice purely personal and little capable of delegation in any respect, while the young surgeon, for example, is assisted in tiding over his years of waiting by acting as assistant at the operations of senior colleagues of established reputation.

Humanity dearly loves a grumble, and it is seldom that a man is heard praising in public the occupation which gives him his livelihood, though as likely as not he will put his son to the same task in spite of his censure of it. We need not, therefore, take too seriously the gloomy prognostics so freely bandied concerning the future of the medical profession. It is undoubtedly a hard and often anxious life, and its financial rewards are not always what might be wished; but for all its devotees it has many returns which cannot be expressed in pounds, shillings and pence, while to not a few of the more able and lucky it yields a substantial fortune. Moreover, it gives to those who follow it an honourable position, and one whose dignity is gradually advancing with its progressive success both in prevention and cure. No position in life is devoid of drawbacks, and, all things considered, medicine shows as large a balance of advantage as any other profession.

